# Targeting MAGE-C1/CT7 Expression Increases Cell Sensitivity to the Proteasome Inhibitor Bortezomib in Multiple Myeloma Cell Lines

**DOI:** 10.1371/journal.pone.0027707

**Published:** 2011-11-16

**Authors:** Fabricio de Carvalho, Erico T. Costa, Anamaria A. Camargo, Juliana C. Gregorio, Cibele Masotti, Valeria C.C. Andrade, Bryan E. Strauss, Otavia L. Caballero, Djordje Atanackovic, Gisele W.B. Colleoni

**Affiliations:** 1 Disciplina de Hematologia e Hemoterapia, Universidade Federal de São Paulo, São Paulo, Brazil; 2 Laboratory of Molecular Biology and Genomics, Ludwig Institute for Cancer Research, São Paulo, Brazil; 3 Recepta Biopharma, Ludwig Institute for Cancer Research, São Paulo, Brazil; 4 Setor de Vetores Virais, Faculdade de Medicina, Laboratório de Genética e Cardiologia Molecular, InCor, São Paulo, Brazil; 5 Ludwig Collaborative Group, Department of Neurosurgery, Johns Hopkins University School of Medicine, Baltimore, Maryland, United States of America; 6 Department of Medicine II, Oncology/Hematology/Stem Cell Transplantation, University Medical Center Hamburg-Eppendorf, Hamburg, Germany; Universidade de Sao Paulo, Brazil

## Abstract

The *MAGE-C1/CT7* encodes a cancer/testis antigen (CTA), is located on the chromosomal region Xq26–27 and is highly polymorphic in humans. *MAGE-C1/CT7* is frequently expressed in multiple myeloma (MM) that may be a potential target for immunotherapy in this still incurable disease. MAGEC1/CT7 expression is restricted to malignant plasma cells and it has been suggested that *MAGE-C1/CT7* might play a pathogenic role in MM; however, the exact function this protein in the pathophysiology of MM is not yet understood. Our objectives were (1) to clarify the role of *MAGE-C1/CT7* in the control of cellular proliferation and cell cycle in myeloma and (2) to evaluate the impact of silencing *MAGE-C1/CT7* on myeloma cells treated with bortezomib. Myeloma cell line SKO-007 was transduced for stable expression of shRNA-*MAGE-C1/CT7*. Downregulation of MAGE-C1/CT7 was confirmed by real time quantitative PCR and western blot. Functional assays included cell proliferation, cell invasion, cell cycle analysis and apoptosis. Western blot showed a 70–80% decrease in MAGE-C1/CT7 protein expression in inhibited cells (shRNA-*MAGE-C1/CT7*) when compared with controls. Functional assays did not indicate a difference in cell proliferation and DNA synthesis when inhibited cells were compared with controls. However, we found a decreased percentage of cells in the G2/M phase of the cell cycle among inhibited cells, but not in the controls (p<0.05). When myeloma cells were treated with bortezomib, we observed a 48% reduction of cells in the G2/M phase among inhibited cells while controls showed 13% (empty vector) and 9% (ineffective shRNA) reduction, respectively (p<0.01). Furthermore, inhibited cells treated with bortezomib showed an increased percentage of apoptotic cells (Annexin V+/PI-) in comparison with bortezomib-treated controls (p<0.001). We found that *MAGE-C1/CT7* protects SKO-007 cells against bortezomib-induced apoptosis. Therefore, we could speculate that *MAGE-C1/CT7* gene therapy could be a strategy for future therapies in MM, in particular in combination with proteasome inhibitors.

## Introduction

Multiple Myeloma (MM) is the second most frequent hematological malignancy. It is a cancer characterized by the infiltration and growth of malignant monoclonal plasma cells in the bone marrow microenvironment, presence of monoclonal immunoglobulin in the blood and/or urine, and lytic bone lesions [1]–[5]. The characterization of the mechanisms responsible for expansion MM cells is difficult due to many genetic alterations identified in malignant plasma cells as well as changes in bone marrow microenvironment leading to tumor growth and immune system failure [6]. MM remains an incurable disease despite all current treatments with median survival varying from 3 to 5 years [7]–[9].

Currently three drugs are being widely used in the treatment of patients with MM: bortezomib (Velcade; Millennium Pharmaceuticals, Inc., Cambridge, MA, Johnson and Johnson Pharmaceuticals Research and Development & L.L.C., Raritan, NJ), as part of the first line therapy in candidates for autologous transplantation or for those who have poor prognostic factors; thalidomide (Thalomid; Celgene Corp., Summit, NJ), used in combination with dexamethasone, was approved in 2006 for the treatment of newly diagnosed MM; lenalidomide (Revlimid; Celgene Corp., Summit NJ) thalidomide analogue, used in combination with dexamethasone, and recommended as part of the first line treatment for patients who present no poor prognostic factors [7].

Cancer/testis antigens (CTAs) are tumor-associated genes originally discovered in patients with malignant melanoma, with the ability to elicit cytotoxic T cells and humoral immunity [10]–[14]. These antigens are expressed in a broad range of human tumors, but in normal tissues, their expression is limited to testis, fetal ovary, and occasionally placenta, and confined to immature cells such as spermatogonia, oogonia, and trophoblasts [15]–[19]. CTAs are grouped into more than 40 distinct families based on their strongly immunogenic properties, expression profiles and by bioinformatics methods [19]–[21]. Many CTAs are considered attractive targets for cancer immunotherapy because the gonads are immune protected organs and anti-CTA immune responses will therefore target tumors specifically [21], [22]. It is possible that CTA have specific biological roles in different tumor types, but their exact function in tumorigenesis and/or promotion of the malignant phenotype remain to be elucidated [23], [24].

The *MAGE-C1/CT7* CTA gene is located on the chromosomal region Xq26-27 and was identified simultaneously by representational difference analysis (RDA) and serological analysis of recombinant cDNA expression libraries (SEREX) [25], [26]. *MAGE-C1/CT7* is highly polymorphic in humans, due to variations in the number of repeat units between different alleles. The function of its protein is not yet understood but it seems to be associated with a more aggressive clinical behavior in some human epithelial cancers [25], [27].

In multiple myeloma (MM), *MAGE-C1/CT7* expression is restricted to malignant plasma cells [10], [28]. Andrade *et al.* [29] studied bone marrow aspirates obtained at diagnosis of MM and observed *MAGE-C1/CT7* expression in 77% of all MM patients and one of three monoclonal gammopathy of undetermined significance (MGUS) cases analyzed. Atanackovic *et al.* [21] have suggested that especially *MAGE-C1/CT7* might promote the progression of MM, since it seems to play a role as a ‘gatekeeper’ gene for other CTA antigens and can be associated with a more aggressive phenotype.

Prompted by the hypothesis that the *MAGE-C1/CT7* gene could have an important biological role in MM tumorigenesis, we planned: (1) to identify MM cell lines with *MAGEC1/CT7* expression, (2) to obtain a stable and efficient silencing of *MAGE-C1/CT7* gene by small hairpin RNA (shRNA) in a *MAGE-C1/CT7*-positive myeloma cell line to perform functional studies, and (3) to evaluate the impact of silencing *MAGE-C1/CT7* on cells treated with novel proteasome inhibitor anti-myeloma agent bortezomib.

## Results

### Expression pattern of MAGE-C1/CT7 in SKO-007, U266, SK-MM-2 and RPMI-8226 analyzed cell lines

In this functional study, we investigated the level of *MAGE-C1/CT7* expression in four MM cell lines (SKO-007, U266, SK-MM-2 and RPMI-8226) by RT-PCR.

**Figure 1:**
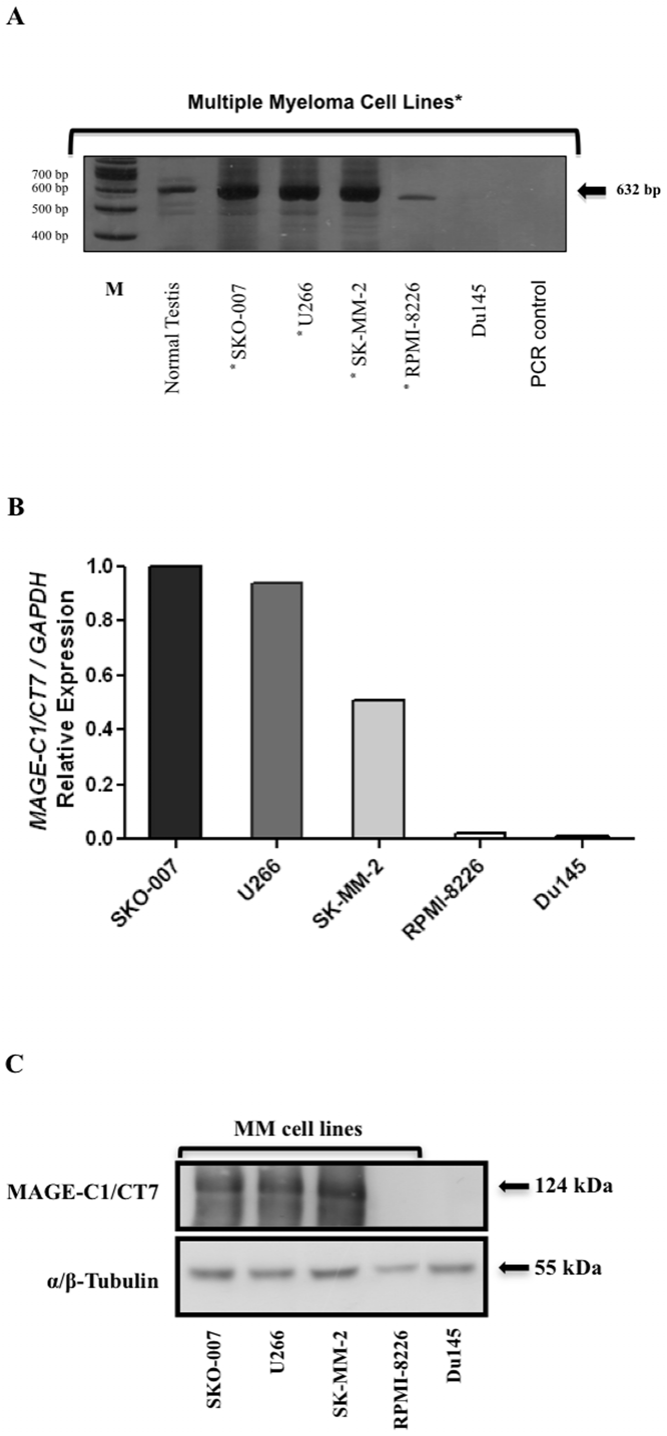
*MAGE-C1/CT7* expression in Multiple Myeloma (MM) cell lines. A) RT-PCR products in 8% polyacrylamide gel electrophoresis and visualized by silver staining. RT-PCR products from cDNAs demonstrating qualitative difference in mRNA expression of *MAGE-C1/CT7* in normal testis and four multiple myeloma cell lines (SKO-007, U266, SK-MM-2 and RPMI-8226). PCR control  =  PCR amplification without cDNA template to rule out contamination. M = 100 bp ladder (Invitrogen). B) Quantitative expression of *MAGE-C1/CT7* in four MM cell lines (SKO-007, U266, SK-MM-2, RPMI-8226) by SYBR green real time quantitative PCR (qPCR). qPCR was performed with cDNA made from MM cell lines using optimized gene-specific primers to analyze mRNA expression levels of *MAGE-C1/CT7* and normalized with *GAPDH* expression in corresponding MM cell lines. Bars represent the levels of relative *MAGE-C1/CT7* expression in each MM cell line. The convectional RT-PCR and SYBR green real time quantitative PCR (qPCR) were performed as described in Materials and Methods section. Du145 prostate cell line was used as negative control for *MAGE-C1/CT7* expression.

All four cell lines expressed *MAGE-C1/CT7* as revealed by RT-PCR, however, RPMI-8226 showed a less intense band in gel electrophoresis (Fig. 1A). This result was confirmed by real-time quantitative PCR [qPCR] (Fig. 1B) and western blot (Fig. 1C). The expression level of *MAGE-C1/CT7* was higher in cell line SKO-007 than in any other cell line [U266, SK-MM-2 and RPMI-8226] (Fig. 1B). Therefore, we chose SKO-007 cells for silencing of *MAGE-C1/CT7* expression using interfering RNA (RNAi).

**Figure 2:**
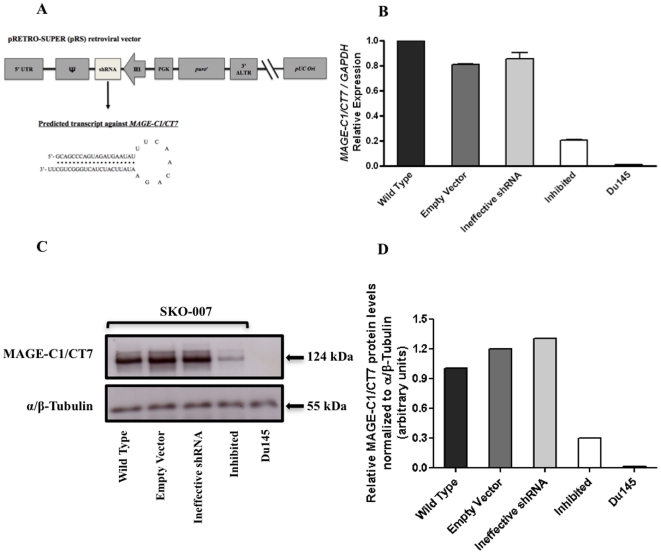
Silencing of *MAGE-C1/CT7* expression in SKO-007 MM cell line transduced with the pRS-shRNA-*MAGE-C1/CT7* construct. Short hairpin RNA (shRNA) constructs against *MAGE-C1/CT7* was stably transduced in MM cell line SKO-007 and effective knockdown was confirmed by qPCR and western blot. A) Schematic representation of the pRETRO-SUPER (pRS) retroviral vector used for silencing of shRNA-*MSAGE-C1/CT7* transcribed from the H1-RNA promoter. B) *MAGE-C1/CT7* expression was 4-5-fold lower in inhibited cells when compared with wild type, empty vector and ineffective shRNA control cells. Du145 prostate cell line was used as negative control of *MAGE-C1/CT7*. C) Western blot using anti-MAGE-C1/CT7 monoclonal antibody (clone CT7.33). α/β-Tubulin protein was used as an internal control. Notably, wild type, empty vector and ineffective shRNA control cells showed constitutive expression of MAGE-C1/CT7 protein. *M*AGE-C1/CT7 protein expression was approximately four times lower in inhibited cells when compared with wild type, empty vector and ineffective shRNA control cells [124 kDa - MAGE-C1/CT7 protein; 55 kDa - α/β-Tubulin protein]. D) Inhibited cells transduced with pRS-shRNA-*MAGE-C1/CT7* construct had an approximately 4-fold (70–80%) decrease in MAGE-C1/CT7 protein expression when compared with control cells. Bars represent the densitometric analysis of protein bands normalized to α/β-Tubulin bands presents in Fig. 2C.

We used a short hairpin RNA (shRNA) specific for *MAGE-C1/CT7* that was previously inserted in the pRETRO-SUPER [pRS] retroviral vector (Fig. 2A). The pRS-shRNA-*MAGE-C1/CT7* construct was co-transfected with pCL-amphotropic packing vector into HEK293T cells to produce virus particles. Virus particles with shRNA-*MAGE-C1/CT7* were transduced and selected in myeloma cell line SKO-007. Cell line SKO-007 was divided into three derivatives: (1) empty vector (pRS), (2) ineffective shRNA (‘scramble’, antisense strand deleted – GC bases), and (3) inhibited (shRNA-*MAGE-C1/CT7*) cells. Analyzing *MAGE-C1/CT7* by qPCR in all three derivatives and wild-type cells we found a 70–80% downregulation of *MAGE-C1/CT7* mRNA expression in inhibited (shRNA-*MAGE-C1/CT7*) cells when compared to controls (Fig. 2B). This finding was paralleled by analyses performed on the protein level using western blot (Fig. 2C-D).

### MAGE-C1/CT7 is not related to cell proliferation and invasion in myeloma cell line SKO-007

**Figure 3:**
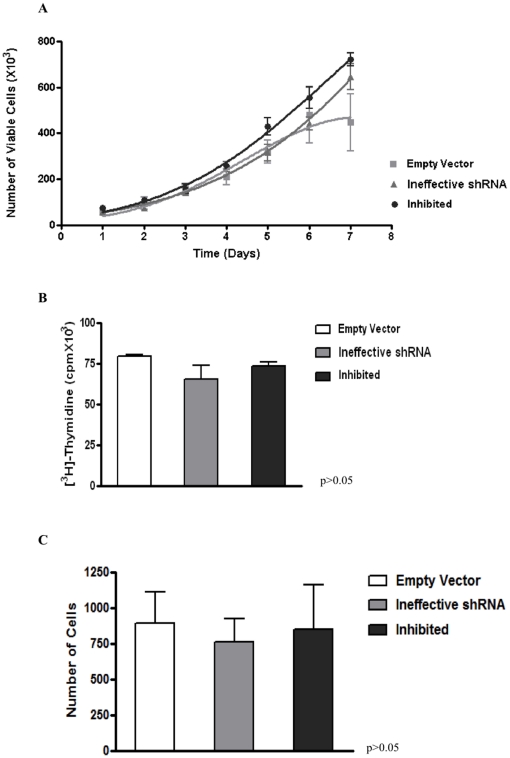
Analysis cell proliferation and invasive potential in myeloma cell line SKO-007. A) Comparison of empty vector (pRS), ineffective (‘scramble’, antisense strand deleted - GC bases) and inhibited shRNA-*MAGE-C1/CT7* (shRNA construct specific for silencing of *MAGE-C1/CT7* expression by RNAi) cells yielded no significant difference between the three growth curves. B) Comparison of thymidine incorporation between empty vector, ineffective shRNA and inhibited shRNA-*MAGE-C1/CT7* cells. There was no significant difference in the DNA synthesis between the three SKO-007 cell derivatives (empty vector, ineffective shRNA and inhibited) by One-Way ANOVA with Tukey multiple comparison test (post test), confirming the result obtained with the growth curves. C) *In vitro* invasion assay using Matrigel for empty vector, ineffective shRNA and inhibited (shRNA-*MAGE-C1/CT7*) cells. There was no significant difference between the number of cells invasion by One-Way ANOVA with Tukey multiple comparison test (post test). All the experiments were independently performed three times and in duplicate.

Cell proliferation of SKO-007 cell derivatives (empty vector, ineffective shRNA and inhibited [shRNA-*MAGE-C1/CT7*]) were assessed by growth curve and [^3^H] thymidine incorporation. SKO-007 inhibited (shRNA-*MAGE-C1/CT7*) growth curves did not show any statistically significant change in cell proliferation when compared with control cells (Fig. 3A). This result suggests that *MAGE-C1/CT7* may not be involved in the proliferation of myeloma cell line SKO-007. Accordingly, no significant difference was seen with regard to DNA synthesis in inhibited (shRNA-*MAGE-C1/CT7*) cells compared with controls (empty vector and ineffective shRNA) in three independent experiments of [^3^H] thymidine incorporation (Fig. 3B).

Finally, we could also not detect any statistically significant change in cellular invasion when we compared inhibited SKO-007 cells (shRNA-*MAGE-C1/CT7*) with controls [empty vector and ineffective shRNA] (Fig. 3C).

### Stable silencing of MAGEC1/CT7 induces changes in cell cycle phases in myeloma cell line SKO-007

**Figure 4:**
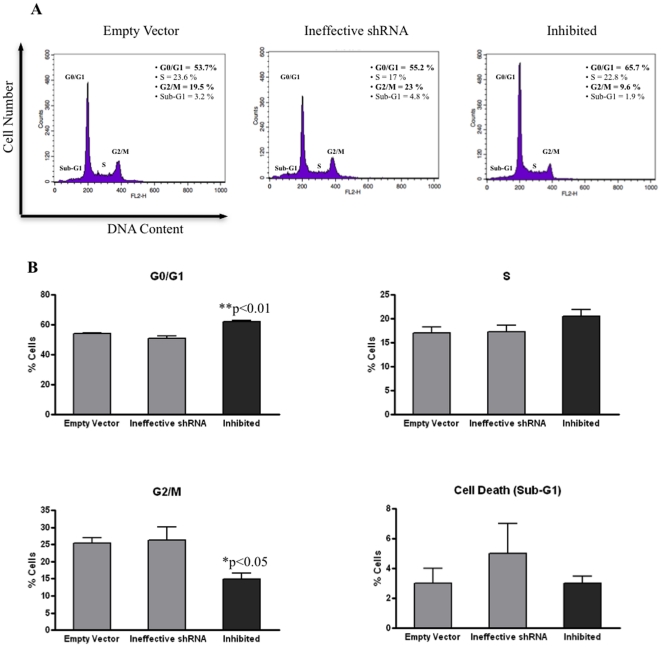
Analysis cell cycle profile by PI staining flow-cytometry in myeloma cell line SKO-007. A) Histograms representing one set of four experiments performed independently. The amount (%) of cells in each cell cycle phase is demonstrated next to each histogram. Inhibited cells showed decrease of cell percentage in G2/M phase and increase of cells in G0/G1 phase when compared with control cells (empty vector and ineffective shRNA). B) Results show mean values (± standard error of means [S.E.M.]) of four independent experiments in duplicate and asterisks (*) indicate statistically significance between inhibited (shRNA-*MAGE-C1/CT7*) cells and controls (empty vector and ineffective shRNA). Inhibited cells showed significant difference in the number of PI stained cells in G0/G1 (**p<0.01) and G2/M (*p<0.05) phases. There was no significant difference (p>0.05) between the three SKO-007 cell derivatives (empty vector, ineffective shRNA, inhibited) in the S phase and cell death (Sub-G1) by One-Way ANOVA with Tukey multiple comparison test (post test). The cells were stained with PI and analyzed for DNA content by flow cytometry as described in Materials and Methods.

In a next step, we asked whether the *MAGE-C1/CT7* gene might be involved in cell cycle regulation because Jungbluth *et al.* [10] had suggested a link between CTA antigen expression and the dysregulation of cell cycle control in MM. Inhibited (shRNA-*MAGE-C1/CT7*) cells and controls (empty vector and ineffective shRNA) were stained with propidium iodide (PI) and the cell cycle status was analyzed by flow cytometry. Results showed a statistically significant increase in the percentage of cells in the G0/G1 phase and a decrease in the percentage of cells in the G2/M phase among inhibited cells compared to controls (Fig. 4). There was no difference regarding the proportion of cells in the S phase of the cell cycle when inhibited cells and controls were compared.

### Bortezomib potentiates the reduction of *MAGE-C1/CT7*-inhibited cells in the G2/M phase

We evaluated the ability of both MA*GE-C1/CT7* silencing and bortezomib to induce alteration in the regulation of the cell cycle in myeloma cell line SKO-007. Empty vector (pRS), ineffective shRNA [both control cells] and inhibited (shRNA-*MAGE-C1/CT7*) cells were treated with 10 nM bortezomib for 48 h, stained with PI and analyzed by flow cytometry.

**Figure 5:**
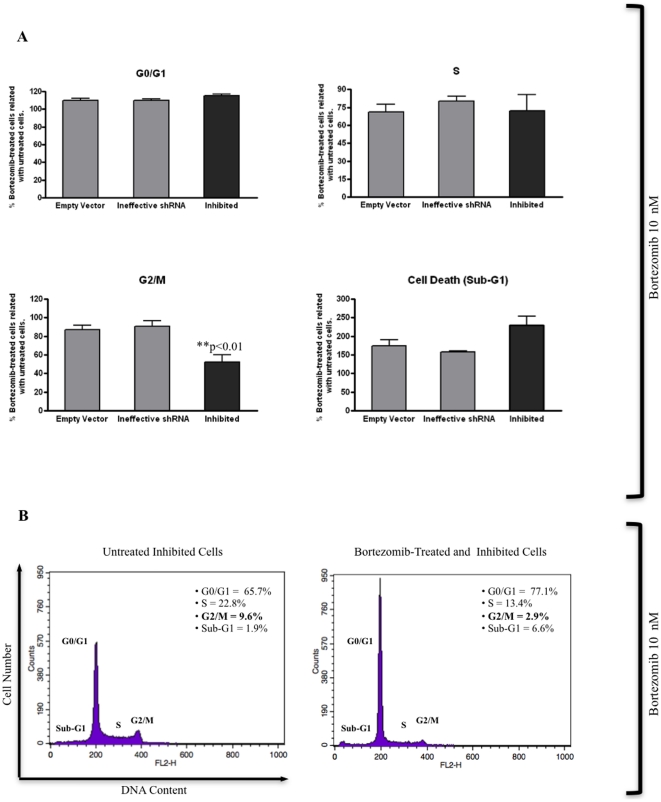
Stable Silencing of *MAGE-C1/CT7* expression in myeloma cell line SKO-007 transduced with pRS-shRNA-*MAGE-C1/CT7* also leads to cell cycle changes when treated with 10 nM bortezomib for 48 h. A) Normalized Results (bortezomib-treated cells vs. untreated cells) show mean values (± standard error of means [S.E.M.]) of four independent experiments in duplicate and asterisks (*) indicate statistically significant between inhibited (shRNA-*MAGE-C1/CT7*) cells and controls (empty vector and ineffective shRNA). In G2/M phase, bortezomib-treated and inhibited cells had lower percentage (48%) of PI stained cells when compared with control cells (empty vector [13%] and ineffective shRNA [9%]) [10 nM bortezomib by 48 h] (**p<0.01). The number of dead cells (Sub-G1) was higher in bortezomib-treated and inhibited (shRNA-*MAGE-C1/CT7*) cells when compared with controls (empty vector and ineffective shRNA), but this difference was not statistically significant. There was also no significant difference (p>0.05) between the three SKO-007 cell derivatives (empty vector, ineffective shRNA, inhibited) in the G0/G1 and S phases of cell cycle by One-Way ANOVA with Tukey multiple comparison test (post test). B) Histograms representing one set of four experiments performed independently. The amount (%) of cells in each cell cycle phase is demonstrated next to each histogram. Bortezomib-treated and inhibited cells showed significant decrease of cell percentage in G2/M phase when compared with untreated counterpart. Cells were stained with PI and analyzed for DNA content by flow cytometry as described in Materials and Methods.

The results were normalized comparing bortezomib-treated with untreated cells (Fig. 5). The G0/G1 and S phase were not significantly altered after treatment with bortezomib among inhibited cells as well as controls. However, we also observed a statistically significant decrease in cells in the G2/M phase among inhibited (shRNA-*MAGE-C1/CT7*) cells, but not in controls, after treatment with bortezomib (Fig. 5A). These combined observations suggest that *MAGE-C1/CT7* gene might plays a role in cell cycle in MM and that silencing of *MAGE-C1/CT7* enhances the anti-myeloma effect of bortezomib.

### MAGE-C1/CT7 silencing increases bortezomib-induced apoptosis in myeloma cell line SKO-007

**Figure 6:**
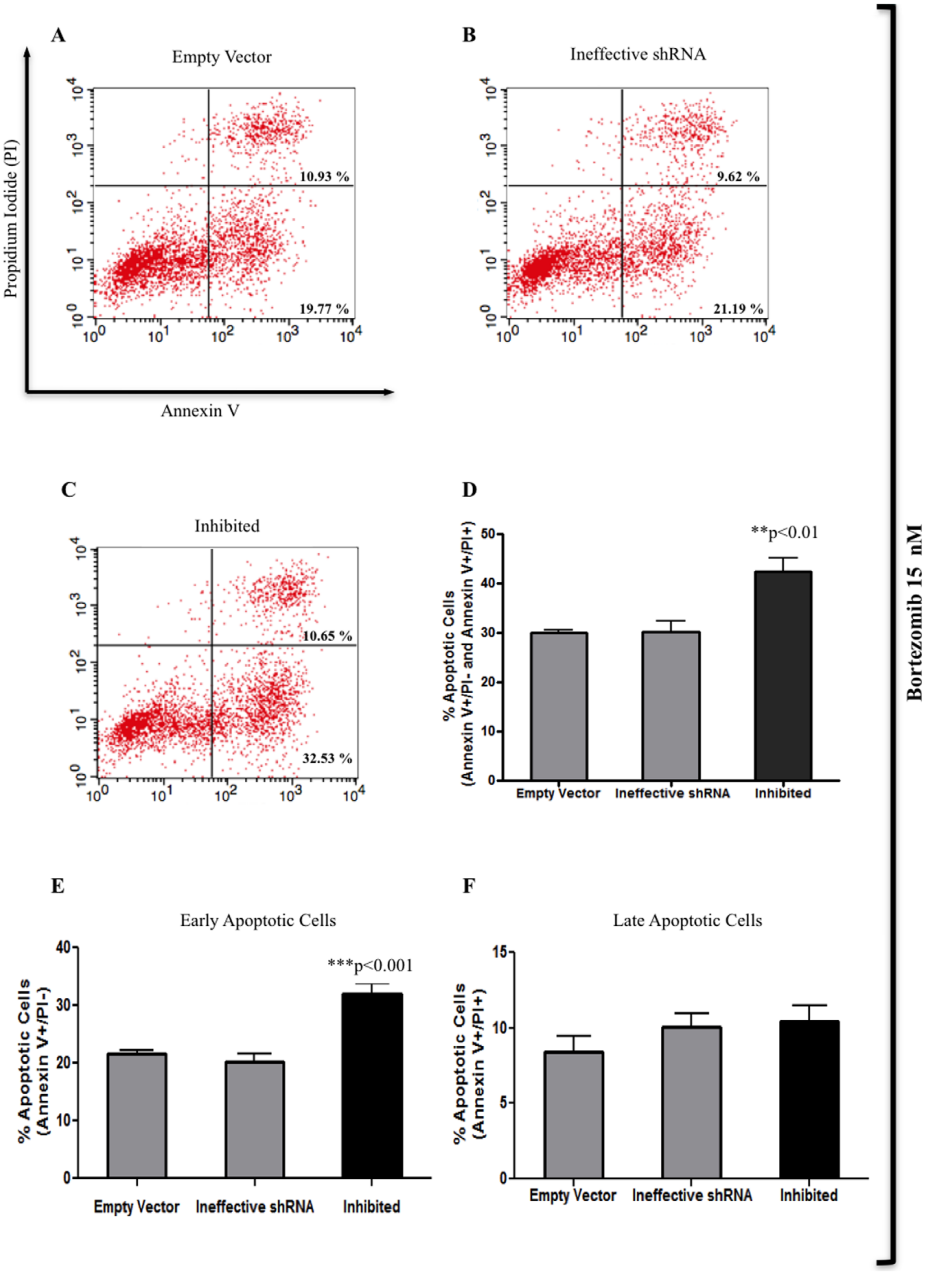
Stable silencing of MAGE-C1/CT7 expression in myeloma cell line SKO-007 leads to increase in apoptotic cells when treated with bortezomib. A-C) Flow cytometry histograms one set of four independent experiments represents Annexin V-FITC staining in *x* axis and PI in *y* axis. The numbers represent the percentage of early (Annexin V+/PI-) [lower right quadrant] and late (Annexin V+/PI+) [upper right quadrant] apoptotic cells in empty vector (pRS), ineffective shRNA and inhibited (shRNA-*MAGE-C1/CT7*) cells treated with 15 nM bortezomib for 48 h. The concentration of 30 nM bortezomib was used as a positive control for Annexin V and PI staining (data not shown). D) Results (bortezomib-treated cells) show mean values (± standard error of means [S.E.M.]) of four independent experiments and asterisks (*) indicate statistically significance between inhibited (shRNA-*MAGE-C1/CT7*) cells and controls (empty vector and ineffective shRNA). Inhibited cells had a statistically significant increase (43%) in the number of apoptotic cells (Annexin V+/PI- and Annexin V+/PI+) compared to control cells (empty vector and ineffective shRNA) (**p<0.01) by One-Way ANOVA with Tukey multiple comparison test (post test). E) Inhibited cells showed statistically significant increase (***p<0.001) in the number of early apoptotic cells (Annexin V+/PI-) compared to control cells by One-Way ANOVA with Tukey multiple comparison test (post test). F) There was no significant increase in the number of late apoptotic cells (Annexin V+/PI+) between the three SKO-007 cell derivatives (empty vector, ineffective shRNA and inhibited) by One-Way ANOVA with Tukey multiple comparison test (post test).

Using Annexin V/PI staining, we next assessed if silencing of *MAGE-C1/CT7* could potentiate bortezomib-induced apoptosis in myeloma cell line SKO-007. Inhibited (shRNA-*MAGE-C1/CT7*) cells and controls were treated with 10 nM and 15 nM of bortezomib for 48 h. 10 nM bortezomib induced apoptosis in three MM cell line SKO-007 derivatives, but no difference was observed in the percentage of apoptotic cells (Annexin V+/PI- or/and Annexin V+/PI+) between inhibited cells and controls (empty vector and ineffective shRNA) (data not shown). However, when inhibited cells and controls (empty vector and ineffective shRNA) were treated with 15 nM bortezomib for 48 h (Fig 6), we observed a 43% increase in apoptosis (Annexin V+/PI- and Annexin V+/PI+) in inhibited (shRNA-*MAGE-C1/CT7*) cells compared to controls (Fig. 6D). Inhibited cells showed statistically significant increase in the number of early apoptotic cells (Annexin V+/PI-) compared to control cells (Fig. 6E) and there was no significant increase in the number of late apoptotic cells/necrotic cells (Annexin V+/PI+) between the three SKO-007 cell derivatives (empty vector, ineffective shRNA and inhibited) (Fig. 6F).

There was no difference in the percentage of apoptotic cells (Annexin V+/PI- and Annexin V+/PI+) between the three SKO-007 cell derivatives (empty vector, ineffective shRNA, inhibited) bortezomib-untreated (Fig. S1). Therefore, it seems that *MAGE-C1/CT7* silencing increases the sensitivity of myeloma cells to bortezomib-induced apoptosis.

## Discussion

We found that silencing of *MAGE-C1/CT7* resulted in a statistically significant increase in the percentage of myeloma cells in G0/G1 phase. On the other hand, silencing of *MAGE-C1/CT7* significantly decreased the number of cells in the G2/M phase of the cell cycle. This decrease was even more pronounced when the inhibited cells were treated with bortezomib, suggesting that myeloma cells with a decreased expression of *MAGE-C1/CT7* might be more susceptible to the effects of bortezomib than controls.

In an attempt to delineate the biological role of *MAGE-C1/CT7* in MM, we first evaluated the expression of CTA mRNA in four MM cell lines (SKO-007, U266, SK-MM-2 and RPMI-8226) by RT-PCR, qPCR and western blot. Myeloma cell line SKO-007 evidenced the highest basal *MAGE-C1/CT7* expression as indicated by qPCR. Therefore, this cell line, which is derived from myeloma line U266, was chosen for subsequent functional analyses. Interestingly, Song *et al.* [30] have previously shown that in the case of myeloma cell line SKO-007, but not U266, cell growth is significantly inhibited following ERK activation in the presence of INFα. This finding suggests that SKO-007 and U266 cells may indeed behave differently with regard to key biological functions.

Analyzing growth curves and [^3^H] thymidine incorporation, we could not detect an influence of *MAGE-C1/CT7* silencing on cell proliferation of inhibited and control cells. This observation would be in line with findings of Atanackovic *et al*. [31] who, performing transient silencing of two CTAs (*MAGE-C1/CT7* and *MAGE-A3*), found only very modest effects on myeloma cell proliferation. On the other hand, Yang *et al.* [32] have previously indicated that silencing of *MAGE* genes might decrease proliferation in neoplastic mast cells *in vitro* and *in vivo*; however, *MAGE-C1/CT7* was not analyzed in that study.

It is important to note that our study is the first to report that myeloma cell line SKO-007 does have invasive potential, providing an important basis for future *in vivo* studies analyzing the potential of anti-myeloma therapies. However, in our current study, using Matrigel we did not detect any difference in cell invasion between inhibited (shRNA-*MAGE-C1/CT7*) cells and controls (empty vector and ineffective shRNA).

A number of studies have indicated that the expression of CTAs in tumor cell lines might induce resistance to chemotherapeutic drugs *in vitro* [33], [34]. Based on these observations, we decided to analyze the cell cycle in inhibited (shRNA-*MAGE-C1/CT7*) cells and controls after treatment with bortezomib. Bortezomib (Velcade) is a boronic acid inhibitor of the 26S ubiquitin/proteasome (formed by the 20S core complex and the 19S regulatory particle) demonstrates potent antitumor activity against several human cancers and has been clinically used in MM treatment [35]–[37]. The ubiquitin/proteasome system has been implicated in cell cycle progression, differentiation, survival, apoptosis, and adhesion [35], [37]. We found that Bortezomib-treated and inhibited (shRNA-*MAGE-C1/CT7*) myeloma cells showed a significant decrease in cells in the G2/M phase when compared with control cells (empty vector and ineffective shRNA). We believe that the bortezomib-treated and inhibited cells in the G2/M phase might represent dying cells because the number of cells did not change in phases G0/G1 and S but increased in Sub-G1 (cell death).

Inhibition of the 26S proteasome by bortezomib results in the accumulation of cyclins A, B, D, E, p21 and p27, thereby disrupting the cell cycle and promoting cell death via multiple pathways [35], [38]. Tamura *et al.* [35] have shown that bortezomib increases the expression levels of cyclin B1, the formation of the cdc1/cyclin B complex, the phosphorylation specific residues on cdc2 and the ubiquitination of cyclin B1 and wee1. These modifications are G2/M-phase-related cell cycle suggesting that bortezomib suppresses the G2/M transition, rather than causing M-phase arrest. Accordingly, Ling *et al.* [39] have shown that bortezomib leads to an increase in the accumulation and activation of G2/M-phase-related cycle regulators cyclin A and cyclin B and to cell cycle blockade at the G2/M phase.

We found more apoptotic cells (Annexin V+/PI-) among bortezomib-treated and inhibited (shRNA-*MAGE-C1/CT7*) myeloma cells than among controls. These results suggest that inhibited cells are more susceptible to bortezomib than control cells and indicate that probably the biological role of *MAGE-C1/CT7* is related to the protection of tumor cells against the effects of cytotoxic drugs. Accordingly, Yang *et al*. [40] have demonstrated that the *MAGE* genes are able to suppress apoptosis and that this event was not affected by caspase inhibitors. Moreover, Atanackovic *et al*. [31] have shown that *MAGE-C1/CT7* and *MAGE-A3* genes play important roles in protecting myeloma cells from spontaneous apoptosis and that silencing these genes further add to the cytotoxic effects of anti-myeloma agents.

In conclusion, our study was the first to achieve stable silencing of *MAGE-C1/CT7* and suggests that this CTA gene might play a role in cell cycle. Furthermore, our findings indicate that *MAGE-C1/CT7 is* involved in protecting myeloma cells against spontaneous as well as drug-induced apoptosis. Silencing of *MAGE-C1/CT7* using shRNA has proven to be a relevant strategy to elucidate the role of this gene, and maybe other CT genes, in myeloma tumorigenesis. Most importantly, we could speculate that targeting *MAGE-C1/CT7* might represent a valuable therapeutic option for myeloma, in particular when applied in combination with proteasome inhibitors such as bortezomib.

## Materials and Methods

### Cell culture

The human MM cell lines SKO-007 [41], RPMI-8226 [42] ^,^ U266 [43] and SK-MM-2 [44] were maintained in RPMI 1640 (Gibco Laboratories, Grand Island, NY) supplemented with 10% fetal bovine serum, 1% L-glutamine, 1% NEAA [non-essential amino acids]) and gentamicin. The current study was approved by the Ethics Committee Hospital São Paulo, Universidade Federal de São Paulo, UNIFESP/EPM (#1495/07).

### Antibodies and drug

Anti-MAGE-C1/CT7 (clone CT7.33) [45], monoclonal antibody was a gift from Dr. Otavia L. Caballero. Bortezomib is commercially available (Velcade® - Janssen-Cilag).

### RT-PCR

Total RNA was prepared from cell line pellets using TRIzol (Invitrogen, Carlsbad, CA, USA) according to the manufacturer's instructions. Two micrograms of total RNA were reverse-transcribed with SuperScript III Reverse Transcriptase (Invitrogen). *MAGE-C1/CT7* was analyzed by RT-PCR and 8% polyacrylamide gel electrophoresis and visualized by silver staining. Normal testis was used as template for positive control in all RT-PCR reactions. PCR reactions were performed using Platinum Taq DNA Polymerase (Invitrogen). The PCR steps performed in an Applied Biosystems GeneAmp PCR 9700 thermocycler, and cycle conditions are initial denaturation at 94°C for 2 min, 35 cycles of denaturation 45 s at 94°C, annealing 63 °C, extending 1 min at 72°C, and final extending at 72°C for 7 min. The sequences of *MAGE-C1/CT7* primers were: forward 5′-GACGAGGATCGTCTCAGGTCAGC-3′ and reverse 5′- ACATCCTCACCCTCA GGAGGG-3′ [29].

### SYBR Green Real Time Quantitative PCR

The optimization of real time quantitative PCR (qPCR) reactions was performed following the manufacturer's instructions (PE Applied Biosystems, Foster City, CA, USA), but scaled down to 20 µL per reaction using SYBR Green PCR Master mix (Applied Biosystems), and 5 µL (20 ng of cDNA) of sample. The primers (Integrated DNA Technologies, IDT, USA) used for *MAGE-C1/CT7* were forward 5′-GAGCTGTAAGCCGGCCTTT-3′ and reverse 5′-TCCCAGCAGTAGGCATATCCTT-3′. *GAPDH* was used as an endogenous control gene for transcription reactions (*GAPDH* forward 5′-GTCCACTGGCGTCTTCACCA-3′ and reverse 5′-GTGGCAGTGATGGCATGGAC-3′). qPCR was performed using an ABI 7300 Sequence Detection System (PE Applied Biosystems) and universal cycling conditions (2 min at 50°C, 10 min at 95°C, 40 cycles of 15 s at 95°C, and 1 min at 60°C). Calculations were made using the comparative CT (2^-ΔΔCT^) between target and constitutive genes. Dissociation curves were recorded after each run to distinguish the main qPCR products from primer dimers, and the products were also analyzed by 8% polyacrylamide gel electrophoresis and visualized by silver staining. All qPCR reactions were performed in duplicate.

### Western Blot

Extracts from 5×10^5^ cells were prepared in 2X lysis buffer (50 mM Tris-Cl pH 6.8, 2% SDS, 10% glycerol, and 0.1% bromophenol blue), incubated at 95°C for 5 min, centrifuged at 4°C for 10 min at 12.000 rpm. Protein lysates were separated on 5% (MAGE-C1/CT7) or 10% (α/β tubulin control) polyacrylamide gels (SDS-PAGE) and electro-transferred to polyvinylidene difluoride (PVDF) membrane (Amersham Hybond-P, GE Healthcare, Buckinghamshire, UK). For MAGE-C1/CT7 protein, the membranes were blocked by incubation in PBST (1X PBS, 0.1% Tween 20) with 3% bovine serum albumin (BSA) for 1 h, and then incubated with the primary antibody (0.5 µg/mL, clone CT7.33) overnight at 4°C in PBST with 3% BSA. After washing three times in PBST for 10 min each, the membrane was incubated with peroxidase-conjugated anti-mouse IgG (dilution of 1∶5,000) for 1 h at room temperature. For α/β tubulin protein, the membrane was blocked by incubation in TBST (Tris-buffered saline [1X TBS], 0.1% Tween 20) with 5% non-fat dry milk for 1 h, and then incubated with the primary antibody (dilution of 1∶1,000) overnight at 4°C in TBST with 5% non-fat dry milk. After washing three times in TBST for 10 min each, the membranes were incubated with peroxidase-conjugated anti-rabbit IgG (dilution of 1∶4,000) for 1h at room temperature. ECL Western Blotting Detection Reagent (Amersham, GE Healthcare, Buckinghamshire, UK) was used to detect antibody binding. The antibodies used were a monoclonal anti-MAGE-C1/CT7 (clone CT7.33) for MAGE-C1 protein, and a rabbit polyclonal anti-α/β tubulin antibody. WBs made for MAGE-C1/CT7 protein and loading control (α/β tubulin) were always performed in parallel and revealed (together) in the same exposure time.

### Vectors

The pCL-amphotropic packaging vector described by Naviaux *et al.* [46] was used for viral production. RNA interference vector construction for *MAGE-C1/CT7* gene silencing includes the retroviral vector pRETRO-SUPER (pRS) [47], which was kindly provided by Dr. Lygia V. Pereira, Department of Genetics and Evolutionary Biology, University of São Paulo, Institute of Bioscience, Brazil. To generate pRS-shRNA-*MAGE-C1/CT7*, the pRETRO-SUPER was digested with *Bgl*II and *Hind*III restriction enzymes. The oligos (forward oligo 5′-gatccccGCAGCCCAGTAGATGAATATttcaagagaATATTCATCTACTGGGCTGCttttggaaa-3′ and reverse oligo 5′-agcttttccaaaaaGCA GCCCAGTAGATGAATATtctcttgaaATATTCATCTACTGGGCTGCggg-3′ were annealed and ligated into the linearized plasmid using T4 DNA ligase (Invitrogen). Chemically competent DH10 *E. coli* was transformed and positive transformants were isolated by ampicillin selection (100 µg/mL) and amplified using standard methods. Presence of insert-containing pRS was confirmed by *Eco*RI/*Hind*III double digest of the plasmid DNA isolated from several bacterial colonies. Plasmid DNA from a positive clone was isolated using a QIAGEN Midi-prep kit (Valencia, CA, USA) and sequenced for additional verification. The 20 nucleotides siRNA sequence for the human *MAGE-C1/CT*7 (GenBank accession number NM_005462.4) was kindly provided by Dr. Otavia L. Caballero.

### Virus Production

Virus production was as described by Bajgelman *et al.* [48]. In 60 mm tissue culture dishes, 7.5×10^5^ HEK293T cells [49], [50] were plated and transfected the next day. To form the precipitate, 10 µg of the plasmid DNA indicated in each experiment plus 10 µg of the packaging vector pCL-amphotropic (prepared with the QIAGEN Plasmid Midi Kit) was mixed with 250 µL of 0.25 M CaCl_2_, then added drop-wise while vortexing to 250 µL of 2X HBS (40 mM Hepes, 2.8 mM Na_2_HPO_4_, 274 mM NaCl), pH 7.05. Precipitate was allowed to form for 10 min at room temperature, and then added drop-wise to the HEK293T cells. After 4 h incubation at 37°C, the cells received a 3 min glycerol shock (15% glycerol in 1X PBS), then they were washed once with 1X PBS, and covered with 1.5 mL fresh, complete DMEM. After 24 h of incubation, the virus containing supernatant was collected, centrifuged for 5 min at 1,000 rpm. The supernatant was removed and stored at −70°C.

### shRNA-*MAGE-C1/CT7* DNA Sequencing Confirmation

Analysis to check the correct sequence of shRNA-*MAGE-C1/CT7* in retroviral vector pRETRO-SUPER (pRS) and confirmation of shRNA-*MAGE-C1/CT7* in the three derivative cells of myeloma cell line SKO-007 (empty vector [pRS] Ineffective shRNA [‘scramble’, antisense strand deleted - GC bases], inhibited [shRNA-*MAGE-C1/CT7*]) by DNA sequencing.

Automated sequencing was Carried Out with the BigDye Terminator Cycle Sequencing Ready Reaction Kit (Perkin Elmer Applied Biosystems, Foster City, CA) using an ABI Prism 3130 DNA confirmation of shRNA-*MAGE-C1/CT7* and therefore there is not need to send it to GeneBank.

### Retrovirus Transduction of shRNA-*MAGE-C1/CT7*

On the day of transduction, SKO-007 cells were transduced (1×10^6^ cells seeded) in 48-well plate along with recombinant retrovirus encoding for shRNA against *MAGE-C1/CT7* at MOI of 1 (multiplicity of infection) in serum-free growth medium containing 8 µg/mL polybrene at 37°C and 5% CO_2_. The cells were incubated for 8 h and then cultured continuously for 3 weeks in the presence of 2.0 µg/mL puromycin (selection for stable expression of shRNA-*MAGE-C1/CT7*) in RPMI 1640 supplemented with 10% FBS, 1% L-glutamine, and 1% NEAA.

### Growth Curve

We calculated the growth rate of cell lines by counting the total number of cells in duplicate wells every day, for 7 days. 1×10^5^ cells in 2 ml RPMI 1640 supplemented with 10% FBS, 1% L-glutamine, and 1% NEAA were seeded into flat-bottomed 6-well plastic culture plate (Corning Costar, Corning, NY). Cell viability was determined by using trypan blue exclusion (Sigma, Saint Louis, Missouri). Three separate experiments were performed in duplicate.

### [^3^H] Thymidine Incorporation

Proliferation assay was performed as described earlier [51], with some modifications. Cells were seeded into flat-bottomed, 24-well plastic culture plate (Corning Costar, Corning, NY, USA) at a density of 5×10^5^ cells/well in 1 mL of complete medium for 24 h. Cells were incubated with ‘hot’ methyl-[^3^H]-thymidine (final concentration of 1.5 µCi) and ‘cold’ thymidine (final concentration of 10^−8^ M) for 18 h. After the incorporation period, cells were transferred to 2 mL Costar microcentrifuge tubes (Corning Costar, Corning, NY, USA) and centrifugated for 2 minutes at 2,000 rpm. After removal of the supernatant, 0.5 mL cold 10% TCA (trichloroacetic acid) were added and incubated for 10 min at room temperature, followed of the centrifugation at 2000 rpm for 2 min. The supernatant was removed and pellets were dissolved in 100 µL 0.5 N NaOH and precipitated. A cellulose filter [1.8 cm×0.8 mm] (3M Company, St. Paul, MN, USA) was added to each microcentrifuge tube. Each cellulose filter was washed once with 10% TCA, twice with 70% ethanol and once with 100% acetone. The cellulose filters were dried for 2 h at 50°C from drying. After drying, the cellulose filter was added to scintillation tubes with 2 mL scintillation solution (POP, POPOP, Toluene and Triton X-100). The radioactivity incorporated into DNA counted was quantified in an LS 6500 Multi-purpose scintillation counter (Beckman Coulter, Fullerton, CA, USA). Experiments were performed at least three times in duplicate.

### Transwell Invasion Assay

*In vitro* invasion assays were carried out using 6.5 mm Transwell membranes (Corning Incorporated, Corning, NY, USA) to measure tumor invasion. The Matrigel invasion chambers were prepared at 1∶3 dilution and incubated for 1 h at 37°C, 5% CO_2_. Cells were washed with 1X PBS, resuspended in 0.1% fetal bovine serum (FBS)-RPMI 1640 and 1×10^5^ cells (80 µL) were added to the Matrigel-coated upper chamber. RPMI-1640 culture medium (0,6 mL) containing 20% FBS was placed in the lower compartment of the chemotaxis chamber to serve as a source of chemoattractants. The 24-well plastic culture plate was incubated at 37°C, 5% CO_2_ for 36 h. After incubation, the non-invading cells and the Matrigel were removed. The invading cells were quantified using flow cytometry BD FACSCalibur (Becton Dickinson, Franklin Lakes, NJ, USA). Three independent experiments were performed in duplicate.

### Analysis of Cell Cycle using PI Staining

Briefly, 0.5×10^6^ cells were cultured in a 24-well plate (Corning Costar, Corning, NY, USA) for 48 h at 37°C, 5% CO_2_, with 1 mL complete medium (RPMI 1640 supplemented with 10% FBS, 1% L-glutamine, and 1% non-essential amino acids) with or without 10 nM bortezomib (Velcade) [31]. Cells were harvested, centrifuged at 2000 rpm for 2 min resuspended in 0.5 mL of hypotonic solution of PI [0.6 mL of 10 mg/mL PI; 100 µL of 20 mg/mL RNase; 0.058 g of NaCl, 0.121 g of Trisma base, and 0.1 mL of NP40; volume to 100 mL, pH 8.0] and incubated overnight at 4°C in the dark to stain nuclear DNA [52]. The cells were centrifuged for 2 min at 2,000 rpm, and resuspended in 0.5 mL 1X PBS. We identified percentages of cells in G1, S, and G2/M phases of the cell cycle by flow cytometry (BD FACSCalibur) excluding cell doublets. Analyses were performed using BD CELLQuest^TM^ Pro software version 3.3 (Becton Dickinson). Each analysis was performed using at least 20,000 events. Four independent experiments were performed in duplicate.

### Detection of Apoptosis via FITC-Annexin V/PI Staining

Briefly, 1×10^6^ cells were cultured in 12-well plate (Corning Costar, Corning, NY, USA) for 48 h at 37°C, 5% CO_2_, with 1 mL complete medium (RPMI 1640 supplemented with 10% FBS, 1% L-glutamine, and 1% non-essential amino acids) with or without 10 nM and 15 nM bortezomib (Velcade). 1×10^5^ cells of each plate were collected and resuspended in 100 µL 1 X Annexin V Binding Buffer (BD Biosciences, San Jose, CA, USA). 2 µL FITC-Annexin V (BD Biosciences) [53] were added as well as 10 µL PI (Sigma, Saint Louis, Missouri, USA) staining to a final concentration of 5 µg/mL and the cells were incubated at room temperature for 15 min in the dark. Then, 400 µL of Annexin V binding buffer were added and flow cytometry was performed using a BD FACSCalibur flow cytometer. Cells were considered to be apoptotic if they were Annexin V+/PI- (early apoptotic) and Annexin V+/PI+ (late apoptotic). Each analysis was performed using at least 20,000 events. Four independent experiments were performed.

### Statistical Analysis

Statistical analyses were performed using GraphPad Software. The one-way ANOVA (Tukey HSD, to verify the results) was used to calculate differences between experimental conditions. Data are presented as the means ± S.E.M. (standard error of means) of at least three independent experiments. A p value of less than 0.05 (p<0.05) was considered statistically significant.

## Supporting Information

Figure S1 **Stable silencing of MAGE-C1/CT7 expression in myeloma cell line SKO-007 not leads to increase in apoptotic cells when bortezomib-untreated.** A-C) Flow cytometry histograms one set of four independent experiments represents Annexin V-FITC staining in *x* axis and PI in *y* axis. The numbers represent the percentage of early (Annexin V+/PI-) [lower right quadrant] and late (Annexin V+/PI+) [upper right quadrant] apoptotic cells in empty vector (pRS), ineffective shRNA and inhibited (shRNA-*MAGE-C1/CT7*) cells bortezomib-untreated for 48 h. 30 nM bortezomib was used as a positive control for Annexin V and PI staining (data not shown). D) Results (bortezomib-untreated cells) show mean values (± standard error of means [S.E.M.]) of four independent experiments and asterisks (*) indicate statistically significance between inhibited (shRNA-*MAGE-C1/CT7*) cells and controls (empty vector and ineffective shRNA). Inhibited cells was not observed a statistically significant increase in the number of apoptotic cells (Annexin V+/PI- and Annexin V+/PI+) compared to control cells by One-Way ANOVA with Tukey multiple comparison test (post test). E) Inhibited cells showed no significant increase in the number of early apoptotic cells (Annexin V+/PI-). F) There was no significant increase in the number of late apoptotic cells/necrotic cells (Annexin V+/PI+) between the three SKO-007 cell derivatives (empty vector, ineffective shRNA and inhibited).
(TIFF)
