# Supplementary figures and images for: Targeting MAGE-C1/CT7 Expression Increases Cell Sensitivity to the Proteasome Inhibitor Bortezomib in Multiple Myeloma Cell Lines
Source: PLoS One. 2011 Nov 16;6(11):e27707. doi: 10.1371/journal.pone.0027707 (PMC3218015; doi:10.1371/journal.pone.0027707)

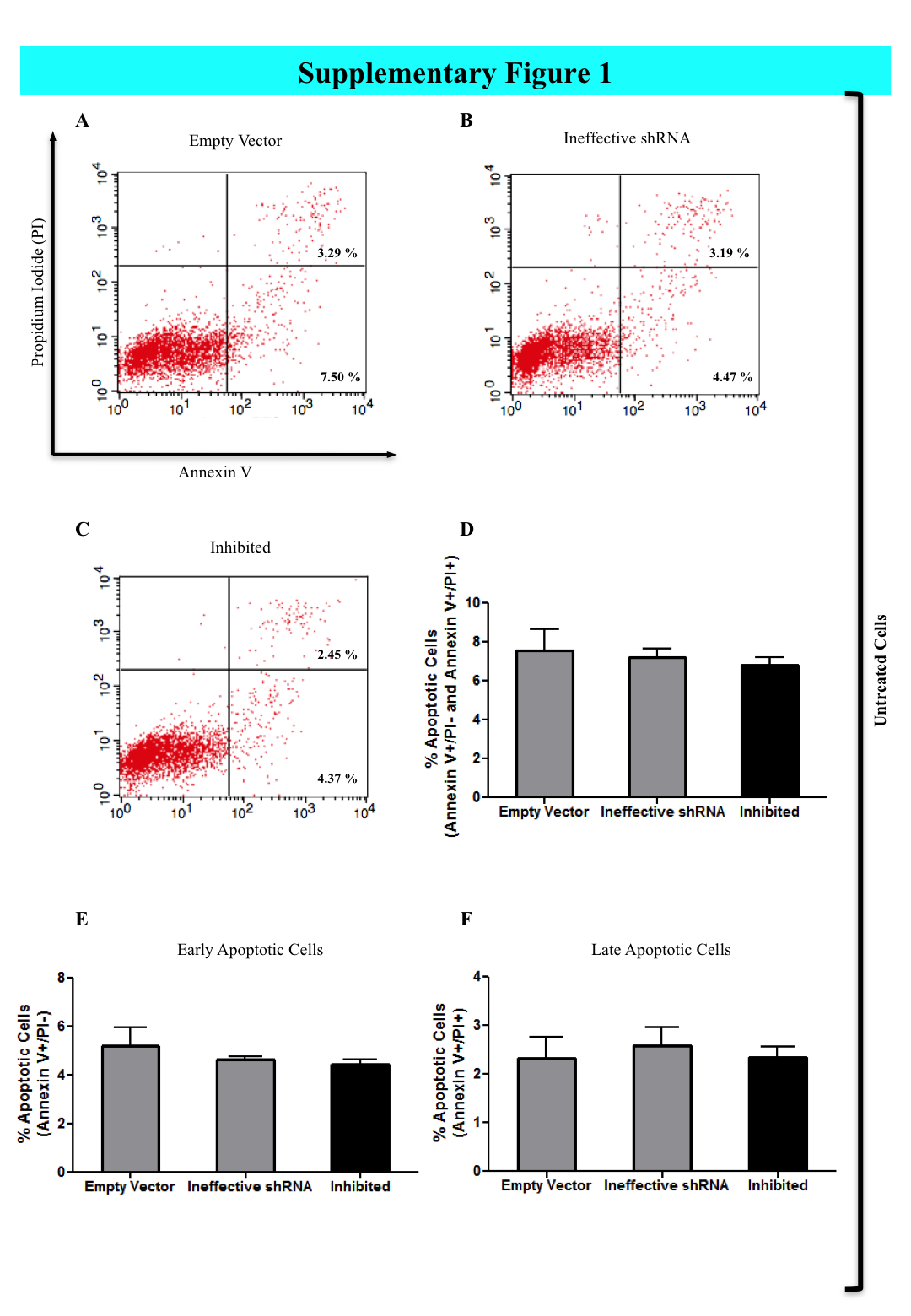

Supplement: Figure S1 — Stable silencing of MAGE-C1/CT7 expression in myeloma cell line SKO-007 not leads to increase in apoptotic cells when bortezomib-untreated. A-C) Flow cytometry histograms one set of four independent experiments represents Annexin V-FITC staining in x axis and PI in y axis. The numbers represent the percentage of early (Annexin V+/PI-) [lower right quadrant] and late (Annexin V+/PI+) [upper right quadrant] apoptotic cells in empty vector (pRS), ineffective shRNA and inhibited (shRNA-MAGE-C1/CT7) cells bortezomib-untreated for 48 h. 30 nM bortezomib was used as a positive control for Annexin V and PI staining (data not shown). D) Results (bortezomib-untreated cells) show mean values (± standard error of means [S.E.M.]) of four independent experiments and asterisks (*) indicate statistically significance between inhibited (shRNA-MAGE-C1/CT7) cells and controls (empty vector and ineffective shRNA). Inhibited cells was not observed a statistically significant increase in the number of apoptotic cells (Annexin V+/PI- and Annexin V+/PI+) compared to control cells by One-Way ANOVA with Tukey multiple comparison test (post test). E) Inhibited cells showed no significant increase in the number of early apoptotic cells (Annexin V+/PI-). F) There was no significant increase in the number of late apoptotic cells/necrotic cells (Annexin V+/PI+) between the three SKO-007 cell derivatives (empty vector, ineffective shRNA and inhibited). (TIFF) [file pone.0027707.s001.tiff]
